# Brief Mindfulness Meditation Induces Gray Matter Changes in a Brain Hub

**DOI:** 10.1155/2020/8830005

**Published:** 2020-11-16

**Authors:** Rongxiang Tang, Karl J. Friston, Yi-Yuan Tang

**Affiliations:** ^1^Department of Psychological and Brain Sciences, Washington University in St. Louis, St. Louis, MO 63105, USA; ^2^Wellcome Centre for Human Neuroimaging, UCL Queen Square Institute of Neurology, University College London, London WC1N 3AR, UK; ^3^Department of Psychological Sciences, Texas Tech University, Lubbock, TX 79409, USA

## Abstract

Previous studies suggest that the practice of long-term (months to years) mindfulness meditation induces structural plasticity in gray matter. However, it remains unknown whether short-term (<30 days) mindfulness meditation in novices could induce similar structural changes. Our previous randomized controlled trials (RCTs) identified white matter changes surrounding the anterior cingulate cortex (ACC) and the posterior cingulate cortex (PCC) within 2 to 4 weeks, following 5-10 h of mindfulness training. Furthermore, these changes were correlated with emotional states in healthy adults. The PCC is a key hub in the functional anatomy implicated in meditation and other perspectival processes. In this longitudinal study using a randomized design, we therefore examined the effect of a 10 h of mindfulness training, the Integrative Body-Mind Training (IBMT) on gray matter volume of the PCC compared to an active control—relaxation training (RT). We found that brief IBMT increased ventral PCC volume and that baseline temperamental trait—an index of individual differences was associated with a reduction in training-induced gray matter increases. Our findings indicate that brief mindfulness meditation induces gray matter plasticity, suggesting that structural changes in ventral PCC—a key hub associated with self-awareness, emotion, cognition, and aging—may have important implications for protecting against mood-related disorders and aging-related cognitive declines.

## 1. Introduction

Decades of scientific research on mindfulness meditation has demonstrated a wide range of positive effects on psychological well-being and related aspects of cognitive function in healthy and clinical populations [1, 2]. Neuroimaging studies speak to the correlates of mindfulness meditation in terms of brain functional and structural plasticity; especially, key brain hubs involved in self-awareness, emotion regulation, and attentional control [1, 3, 4]. Although prior work suggests *functional* changes in both novices with short-term training and experienced meditators with long-term practice, *structural* changes in gray matter have been found mainly in experienced meditators [1, 5]. One preliminary finding indicated changes in gray matter after 2 months of mindfulness-based stress reduction, compared to a waitlist control [6]. However, it remains to be established whether such structural effects of mindfulness training are evident when compared to an active control, using a rigorous randomized design [7–9].

A meta-analysis has identified several brain structures altered by meditation, such as the anterior cingulate cortex (ACC), insula, and hippocampus [5]. However, while both the ACC and insula are important constituents of the salience network implicated in self-awareness and mindfulness meditation [1], the default mode network (DMN) has received less attention in structural studies. The DMN includes the medial prefrontal cortex and posterior cingulate cortex (PCC) and is actively engaged (and affected) in mindfulness as evidenced by functional neuroimaging studies [1, 10, 11]. Prior literature regarding the effect of meditation on the PCC has shown a reduction in its activation during meditation [11], but an increase in its functional connectivity with “task-positive” regions in the executive control network and salience network (e.g., ACC and prefrontal cortex), both at rest and during meditation [11–13]. Because the PCC is commonly implicated in self-referential processing and mind-wandering [14], decreased activation during meditation could be interpreted as reduced mind-wandering, whereas increased coupling with other control-related networks may suggest better self-regulatory function as a result of meditation experiences [11–13]. Therefore, it is plausible that brief or short-term mindfulness could induce not only functional changes in the DMN but also structural plasticity in key nodes or integrative hubs.

Our randomized studies—using a form of mindfulness meditation, the integrative body–mind training (IBMT)—have shown that 5 sessions of IBMT (30 min/session) improved self-control abilities in domains of attention and emotion, as well as increased functional changes in neuronal activity and metabolism in the ACC and PCC [15, 16]. Moreover, 10-20 sessions of IBMT (5-10 h in total) induced white matter plasticity, mainly in white-matter tracts surrounding the ACC and PCC, and the improved mood/affect states were correlated with increased white matter changes surrounding the PCC and other areas [17–20]. These convergent findings suggest that the PCC—a key hub of the DMN [21, 22]—may also undergo structural changes in grey matter following mindfulness.

However, evidence regarding the relationship between mindfulness and the PCC volume has so far been inconclusive. In a study examining trait mindfulness and brain structures, the PCC grey matter volume was found to be negatively related to this tendency to be attentive to and aware of present-moment experiences in everyday life [23]. Conversely, individuals who underwent two months of MBSR showed increased grey matter density in the PCC [6]. Moreover, greater PCC volume in expert meditators was detected compared to controls [24]. Based on the theoretical model regarding the role of PCC [14], as well as preliminary study suggesting mindfulness meditation can induce grey matter improvement [6, 24], we hypothesized that brief IBMT would increase grey matter volume in the ventral PCC, as it is hypothesized to be related to narrow attentional focus both internally and externally, and has also shown greater connectivity with control-related regions in meditation literature [1, 11].

Finally, people differ in their attitudes toward the practice of mindfulness, and that short-term mindfulness practice induces changes in mental state, while long-term practice changes personality or temperamental traits [1, 4, 25]. However, little is known about the role of individual differences (e.g., preexisting differences and traits of temperament) in predisposing to structural plasticity following brief mindfulness. We therefore used the adult temperament questionnaire (ATQ) to assess baseline individual differences [26, 27]. Given that our prior work showed behavioral improvement in emotion or affect was related to increased white matter plasticity surrounding the PCC, we focused on affect-related temperamental traits within the ATQ to examine their relationships with the magnitude of gray matter changes in PCC. We hypothesized that preexisting individual differences in affectivity would predict the degree of gray matter plasticity in PCC following brief IBMT. A significant correlation between baseline temperamental trait and gray matter change would provide important evidence for a role of individual differences in influencing brain structural plasticity following mindfulness.

## 2. Materials and Methods

### 2.1. Participants

Forty-four healthy and meditation-naive college students (*M* = 20.28 years, SD = 1.47 years) were recruited and randomly assigned to either the IBMT group (22 participants, 13 males) or the relaxation group (RT) (22 participants, 14 males). The randomized controlled trial (RCT) was approved by the University of Oregon Institutional Review Board, and informed consent was obtained from each participant. Behavioral and brain measurements included Adult Temperament Questionnaire (ATQ) and functional magnetic resonance imaging (fMRI).

### 2.2. Experiment Design

We used a longitudinal randomized design with an active control RT group and an intervention IBMT group in this study.

### 2.3. Behavioral Measurement

Our previous studies have shown that brief IBMT increases cognition, emotion, and behavior [4, 15, 18, 28–30]. Therefore, we did not conduct these measurements in this study but chose the widely used short form of ATQ with 77 items [26, 27] to examine individual differences in temperament. The ATQ assesses four general constructs (also known as factor scales), including effortful control, negative affect, extraversion/surgency, and orienting sensitivity, developed based on a self-report model of temperament [26, 27]. Our previous randomized studies have shown that the mood/affect states correlate with white matter plasticity following short-term IBMT [18], but a relationship between mood/affect trait of temperament and gray matter plasticity has not been established. Given our hypothesis, we were particularly interested in the positive affect subconstruct of extraversion and nonaggressive subconstructs of negative affect including fear, sadness, and discomfort, which tend to relate more closely to emotion dysregulation and symptoms of mood-related disorders such as depression. Two participants were excluded from further analysis due to incomplete ATQ data.

### 2.4. Structural MRI Data Acquisition

All brain imaging data were collected via a 3 Tesla Siemens scanner. A high resolution (1 × 1 × 1 mm) T1-weighted whole-brain image (with TR = 2500 msec, TE = 4.38 msec, TI = 1100 msec, flip angle = 8°) was acquired for every participant, using a standard magnetization prepared rapid gradient-echo (MPRAGE) sequence. After visual inspection, structural data from four participants were excluded due to structural abnormality or poor data quality from excessive motion.

### 2.5. Statistical Analysis

Statistical analyses were performed by the use of IBM SPSS Statistics 20.0 and Free-Surfer 5.3 (http://surfer.nmr.mgh.harvard.edu/). Structural data were automatically processed using FreeSurfer for cortical reconstruction and segmentation. A standard longitudinal processing pipeline was employed to extract reliable volume estimates [31]. Specifically, an unbiased within-subject template space and image [32] was created using robust, inverse consistent registration [33]. Several processing steps, such as skull stripping, Talairach transforms, atlas registration, and spherical surface maps and parcellations, were then initialized with common information from the within-subject template, significantly increasing reliability and statistical power [31]. The white and pial surfaces were visually inspected and were manually edited to correct for errors when necessary.

The index of symmetrized percent change (spc) is a standard measure for examining longitudinal structural changes in volume and cortical thickness and is sensitive to intervention effects. The spc is the rate of change with respect to the average volume of time point 1 and time point 2: spc = 100∗rate/avg. For longitudinal design, this is also a more robust measure (more statistical power) than the rate of change from time point 1 to time point 2 [31]. The spc has been used widely in studies of mental training, Alzheimer's disease, aging, mood disorders, traumatic brain injury, and other neuropsychiatric disorders [31, 34–40]. To examine whether IBMT and RT groups exhibit differences after 10 h of training, we calculated the spc of bilateral ventral posterior cingulate cortex/isthmus of cingulate (ventral PCC/ISC). The PCC was subdivided into dorsal and ventral portions by the Destrieux cortical parcellation scheme [41]. We are specifically interested in the ventral PCC, defined as the “G_cingul-Post-ventral” in the Destrieux cortial atlas. The spc of bilateral ventral PCC volumes was examined separately for left and right hemispheres. We calculated spc using long_stats_slopes command lines in Free-Surfer, part of the longitudinal processing pipelines. The calculation of spc yielded a single measure for each participant, where positive values indicate increases in volume, and *vice versa* for negative values.

### 2.6. Training Methods

Integrative Body-Mind Training (IBMT) is an open-monitoring mindfulness meditation that mainly involves bodifulness and mindfulness techniques. Bodifulness refers to the gentle adjustment and exercise of body postures with a full awareness, in order to achieve a presence, balance, and integration in our bodies [1, 4, 42, 43]. In each session, guided by an experienced IBMT coach, participants start from bodifulness—the body is naturally relaxed and extended, the mind is calm but alert, and the balanced body postures flow from one to another to promote concentration and mindfulness. IBMT emphasizes the cooperation between body and mind in facilitating and achieving a mindfulness state ecologically. The interaction between mind and body involves both the central nervous system and autonomic nervous system [4, 42]. IBMT stresses no effort to control thoughts but instead encourages a natural state of restful alertness and accepts whatever arises in one's awareness at each moment that facilitates a high degree of awareness of body, mind, and environment [3, 4].

Relaxation training (RT) involves the relaxing of different muscle groups over the face, head, shoulders, arms, legs, chest, back, abdomen, and so on. With eyes closed and in a sequential fashion, one concentrates on the sensation of relaxation, such as the feelings of warmth and heaviness. This progressive training helps the participant achieve physical and mental relaxation and calmness [15]. The participants received 30 min of IBMT, or RT group practice every night for 20 consecutive sessions in lab, for a total of 10 h of training. The participants were not instructed to practice outside of IBMT or RT training.

## 3. Results

### 3.1. Brain Imaging

**Figure 1:**
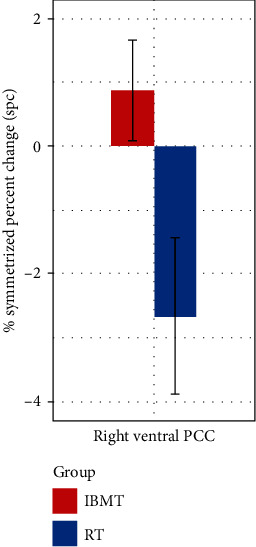
Symmetrized percent change (SPC) of right ventral PCC/ISC. Compared to RT, IBMT induced a significantly higher spc of right ventral PCC/ISC volume.

**Figure 2:**
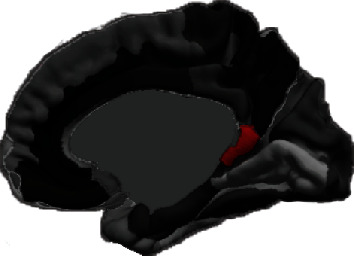
Increased volume of right ventral PCC/ISC. Display of increased volume of ventral PCC/ISC following IBMT.

Before training, the two groups did not differ significantly in terms of bilateral ventral PCC/ISC volumes (independent-samples *t*-tests, *p* > 0.05). To detect longitudinal structural changes using spc, an index sensitive to intervention effects, analysis of covariance (ANCOVA) was conducted between the two groups, with group assignment as the independent variable, while controlling for age and gender as covariates. As hypothesized, we found a significant effect of group on the spc of right ventral PCC/ISC volume *F* (1, 36) = 5.08, *p* = 0.03, such that the IBMT group had a significantly higher spc (*M* = 0.88, SD = 3.50) relative to the RT group (*M* = −2.66, SD = 5.47), shown in Figure 1. The parcellation of bilateral ventral PCC/ISC is shown in Figure 2. A large effect size 0.124 (partial eta-squared) was detected. However, no significant difference was detected for the spc of left ventral PCC/RSC volume between the IBMT and RT group (*p* > 0.05). Additionally, age and gender did not have any significant effect on the spc of bilateral ventral PCC/ISC volumes. We also explored the spc of bilateral dorsal PCC volumes following IBMT but did not find any significant group effects (*p* > 0.05).

### 3.2. Temperamental Traits and Structural Plasticity

**Figure 3:**
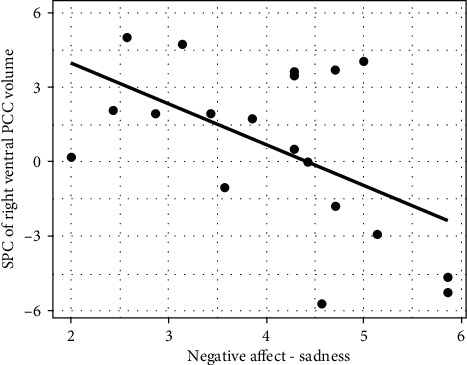
Relationship of negative affect and SPC of right ventral PCC/ISC. Sadness negatively correlated with the spc of right ventral PCC/ISC.

To examine whether temperamental traits are associated with the degree of structural plasticity in ventral PCC/ISC, Pearson's correlations were computed between the spc of right ventral PCC/ISC volume and the four temperamental traits. Sadness was the only subconstruct that showed a significantly negative correlation with the right ventral PCC/ISC spc (*r* = −0.535, *p* < 0.018).  Figure 3 illustrates this negative relationship, such that lower level of unpleasant affect, mood, and energy—related to object or person loss, disappointment, and exposure to suffering—was associated with a greater spc in the right ventral PCC/ISC volume.

## 4. Discussion

Our RCT results demonstrate that 10 h of IBMT induced gray matter changes in ventral PCC/ISC—a brain hub associated with cognition, emotion, and self-related processes (e.g., self-awareness). Moreover, temperamental traits reflecting negative affect predicted the extent of training-induced gray matter volumetric increases in ventral PCC/ISC, suggesting a predisposing role of individual differences in influencing training-induced gray matter plasticity. These findings may have important implications for understanding the pathophysiology of—and monitoring—therapeutic interventions in mood-related disorders and aging-related cognitive decline that often manifest functional and structural abnormalities within these brain regions [1, 14].

Mental training studies such as working memory training, meditation, and yoga have shown both increased and decreased grey matter volume and/or density in different regions throughout the brain [5, 6, 24, 44–46]. In most meditation studies, increases were often detected in areas involving interoceptive awareness and self-regulation, such as the ACC, insular cortex, prefrontal cortex, and sensory cortices [1, 5]. Additionally, hippocampus, a structure critical for memory, has also been shown to increase in volume in long-term meditators [1, 5]. Conversely, reduction in volume was often detected in the amygdala in meditation studies [44, 46], suggesting that this subcortical structure associated with emotion and stress may manifest a different trend of structural plasticity, and may underlie the behavioral reduction of stress reactivity commonly observed in meditators. In the present study, we focused on a brain hub within the default mode network and detected an increase in ventral PCC/ISC volume in the meditation group, which is consistent with the two studies that, respectively, showed greater grey matter density in PCC for novices who underwent meditation training and greater PCC volume in expert meditators relative to controls [6, 24]. According to a theoretical account of the PCC function, the ventral PCC may play a key role in narrow attentional focus [14], which suggests that the increased volume of the ventral PCC may reflect enhanced attentional control following meditation training.

Interestingly, for the relaxation training group, we detected a reduction of grey matter volume in bilateral ventral PCC/ISC. It is important to recognize that the relaxation training has a very different emphasis from the mindfulness training; thus, it is possible that these two forms of training work through different neural mechanisms that led to structural changes in completely different directions. On the other hand, reduction in grey matter volume and density is not uncommon in training studies [44–46], and one possible mechanism of the observed reduction in the relaxation group might be the usage-dependent selective elimination of synapses which helps to sculpt neural circuitry [45]. However, further investigations are needed to fully examine the impact of relaxation training on brain plasticity of the PCC, as well as that of other brain regions. Overall, our finding concerning the meditation group was in line with prior literature that showed structural improvement in the PCC either following short-term or long-term meditation experiences.

Looking more closely at our target region, ventral PCC (vPCC), it includes the ventral subdivision of PCC and isthmus of cingulate (ISC) as defined by the Destrieux cortical atlas. The ISC lies within Brodmann areas 29/30, overlapping the same BA 29/30 with adjacent retrosplenial cortex (RSC). The anatomical location of RSC (BA 29/30) is behind the splenium of the corpus callosum, where RSC directly connects to the vPCC (BA 23) [41, 47–49]. Our previous work showed enhanced white matter connectivity in the vPCC and adjacent ISC/RSC following 5-10 h of IBMT, suggesting a putative relationship between the concurrent structural plasticity of white and grey matter in vPCC and adjacent ISC/RSC following brief mindfulness [4]. A closer examination of these brain regions reveals two important functions: emotion- and cognition-related processes [34, 50–65].

Emotion-related problems and disorders—such as clinical depression and subthreshold depressive symptoms in adults—are related to structural changes in cingulate cortex [34, 53–55]. In a nonclinical sample, one study examined the relationship between depressive symptoms and gray matter volumes in the ACC, PCC, and ISC/RSC and showed that higher scores on the somatic symptoms were related to smaller volume in the PCC and that higher scores on the depressed mood were associated with smaller volume in the ISC/RSC [53]. A further study indicated that unipolar depressed patients had smaller ACC and PCC volumes compared to healthy subjects. Additionally, when patients were divided into currently depressed and remitted subgroups, currently depressed patients had smaller ACC and PCC volumes than the healthy controls [54]. Similarly, one study suggests that the gray matter volumes of PCC and hippocampus are key regions that disambiguate MDD and BD patients from healthy controls [55]. Together, these findings suggest that the PCC and ISC/RSC actively engage in emotional processing and regulation, which may explain why enhanced mood and reduced negative affect are often reported after mindfulness [1]. Increased volume in vPCC and ISC/RSC may be associated with better psychological well-being, which could be one of the mechanisms that underwrites the commonly observed improvement in mood and psychological health following mindfulness [42]. Our findings also provide support for the notion that increasing structural plasticity of vPCC and ISC/RSC may protect individuals against mood-related symptoms, making them less susceptible to emotion-related disorders.

The vPCC and RSC have dense connections to medial temporal lobe, hippocampus, and parahippocampal cortex, which are important areas for memory [47–51]. Growing evidence suggests that decreased PCC activity (e.g., resting-state or task) and metabolism (e.g., glucose metabolic rate) are associated with cognitive decline, mild cognitive impairment (MCI), and Alzheimer's disease (AD) [56–62], which often severely impact memory-related functions. In addition, vPCC also showed associations with MCI/AD, consistent with previous reports concerning the importance of PCC in amyloid deposition with AD—and performance on an episodic memory retrieval task in MCI [63, 64]. Moreover, the locations of amyloid deposition and fMRI activity in both studies were centered on vPCC. Relatedly, greater cortical thickness in PCC is found in high-performing elderly, indicating that greater gray matter in PCC may be a putative signature of optimal aging [65]. This is consistent with the evidence that the PCC may play a direct role in regulating the focus of attention and thus facilitating cognitive performance [4, 14]. There is also support for the roles of vPCC and RSC in internally directed thoughts and cognition such as the retrieval of episodic and semantic memories, imagining, and planning [14, 52]. Lastly, RSC is associated with perspective taking and switching between different frames of reference, which is a capacity that mindfulness practices seek to promote [4, 48].

Previous work showed that five sessions of IBMT improves PCC activity and metabolism, and cognitive function such as attention, creativity, working memory, problem-solving, learning, and self-control capacities [1, 4, 15–18, 28–30]. The present structural finding endorses our functional and behavioral findings, implicating the same brain hub—vPCC and suggests that increased functional and structural plasticity in the PCC may play a key role in the preservation of cognitive capacity and performance in aging population [13, 65]. Increased gray matter in the PCC following 10 h of IBMT in healthy adults may suggest some protection against aging-related processes, such as cognitive decline and MCI [4, 66]. It is worth pointing out that only right vPCC/ISC/RSC was significantly affected by brief IBMT, which is consistent with prior work showing the lateralized function of RSC—the right RSC is more associated with MCI/AD and memory-related impairment than left RSC [52].

Network neuroscience on large-scale (intrinsic) brain networks has indicated an architectural core (i.e., brain hub) in the PCC and parietal cortex, regions with high degree, strength and betweenness centrality, and constitute connector hubs that link major structural modules [22], suggesting that PCC is a key brain hub for efficient information processing—which is implicated in diverse cognitive processes and emotion regulation. Prior work has shown that mindfulness directly changes these mental processes [3, 4]. One emotion regulation strategy, distancing (e.g., reformulating aversive stimuli in a neutral and objective way), is one of mindfulness skills that emphasizes the experience of presence without judgment. Indeed, increased PCC activity and decreased amygdala activity are related to distancing [67], consistent with recent meta-analyses showing that the PCC is one of the main regions implicated in the upregulation and downregulation of emotion [68, 69]. Studies also indicate a role of PCC in conscious awareness, which may explain why enhanced interoceptive awareness is typically observed following mindfulness [1, 3, 70]. Furthermore, PCC has been thought to underpin consciousness, further supporting a role in self-awareness and self-related processing [71–73].

Individual differences in mindfulness training-induced brain structural plasticity are rarely explored [1, 4]. We provide preliminary evidence that temperament traits may play a role in underwriting structural volumetric responses to brief mindfulness. Sadness is one of the negative affects in the ATQ and defined as unpleasant affect/mood, energy related to object/person loss, disappointment, and exposure to suffering. Studies showed that sadness is associated with depressive symptoms [73]. Given the strong functional relevance of vPCC/ISC/RSC in the pathophysiology of depression, it is not surprising that preexisting individual differences in this particular trait could influence subsequent volumetric change. We found that individuals with higher level of sadness either showed less improvement (or indeed reduction) in gray matter volume following mindfulness, suggesting that they were not sensitive to such training and that alternative interventions may be more effective. Crucially, the fact that preexisting temperamental traits were able to predict subsequent gray matter changes suggests that individual differences may play an important role in underlying mechanisms of brain structural plasticity.

Taken together, our results to date suggest it is possible to induce gray matter plasticity in the vPCC and adjacent ISC/RSC following brief mindfulness in novices. However, we do not yet know how long the plasticity will last; this warrants further investigation. Given the relatively small sample size and exploratory nature of the study, the findings need to be validated by future studies. Nevertheless, the present findings may have important implications for protecting individuals against mood-related disorders and aging-related cognitive declines, which exhibit brain functional and structural abnormalities in the vPCC.

## Data Availability

The datasets used to support the findings of this study are available from the corresponding author upon reasonable request.
